# Eukaryotic yeast V_1_-ATPase rotary mechanism insights revealed by high-resolution single-molecule studies

**DOI:** 10.3389/fmolb.2024.1269040

**Published:** 2024-03-19

**Authors:** Seiga Yanagisawa, Zain A. Bukhari, Karlett J. Parra, Wayne D. Frasch

**Affiliations:** ^1^ School of Life Sciences, Arizona State University, Tempe, AZ, United States; ^2^ Department of Biochemistry and Molecular Biology, University of New Mexico School of Medicine, Albuquerque, NM, United States

**Keywords:** eukaryotic V_1_V_O_ ATPase, V_1_-ATPase, single-molecule studies, rotary molecular motor, yeast vacuolar ATPase

## Abstract

Vacuolar ATP-dependent proton pumps (V-ATPases) belong to a super-family of rotary ATPases and ATP synthases. The V_1_ complex consumes ATP to drive rotation of a central rotor that pumps protons across membranes via the V_o_ complex. Eukaryotic V-ATPases are regulated by reversible disassembly of subunit C, V_1_ without C, and V_O._ ATP hydrolysis is thought to generate an unknown rotary state that initiates regulated disassembly. Dissociated V_1_ is inhibited by subunit H that traps it in a specific rotational position. Here, we report the first single-molecule studies with high resolution of time and rotational position of *Saccharomyces cerevisiae* V_1_-ATPase lacking subunits H and C (V_1_ΔHC), which resolves previously elusive dwells and angular velocity changes. Rotation occurred in 120° power strokes separated by dwells comparable to catalytic dwells observed in other rotary ATPases. However, unique V_1_ΔHC rotational features included: 1) faltering power stroke rotation during the first 60°; 2) a dwell often occurring ∼45° after the catalytic dwell, which did not increase in duration at limiting MgATP; 3) a second dwell, ∼2-fold longer occurring 112° that increased in duration and occurrence at limiting MgATP; 4) limiting MgATP-dependent decreases in power stroke angular velocity where dwells were not observed. The results presented here are consistent with MgATP binding to the empty catalytic site at 112° and MgADP released at ∼45°, and provide important new insight concerning the molecular basis for the differences in rotary positions of substrate binding and product release between V-type and F-type ATPases.

## Introduction

Vacuolar H^+^-ATPase (V-ATPase) is an ATP-dependent proton pump that regulates the pH of organelles in eukaryotic cells including Golgi, endosomes, lysosomes, and vacuoles (Kane, 2006). The plasma membranes of certain mammalian cells specialized for proton secretion also contain V-ATPases to aid in proton export from the cell (Breton and Brown, 2013; Collins and Forgac, 2020). V-ATPases are critical for a plethora of cellular processes, including protein processing and secretion, endocytosis and vesicle trafficking, zymogen activation, and autophagy (Kane, 2006; Forgac, 2007). V-ATPases are especially important in human disease (Hinton et al., 2009; Alper, 2010; Breton and Brown, 2013; Hayek et al., 2014; Kartner and Manolson, 2014; Cotter et al., 2015; Cotter et al., 2016; Licon-Munoz et al., 2018).

**FIGURE 1:**
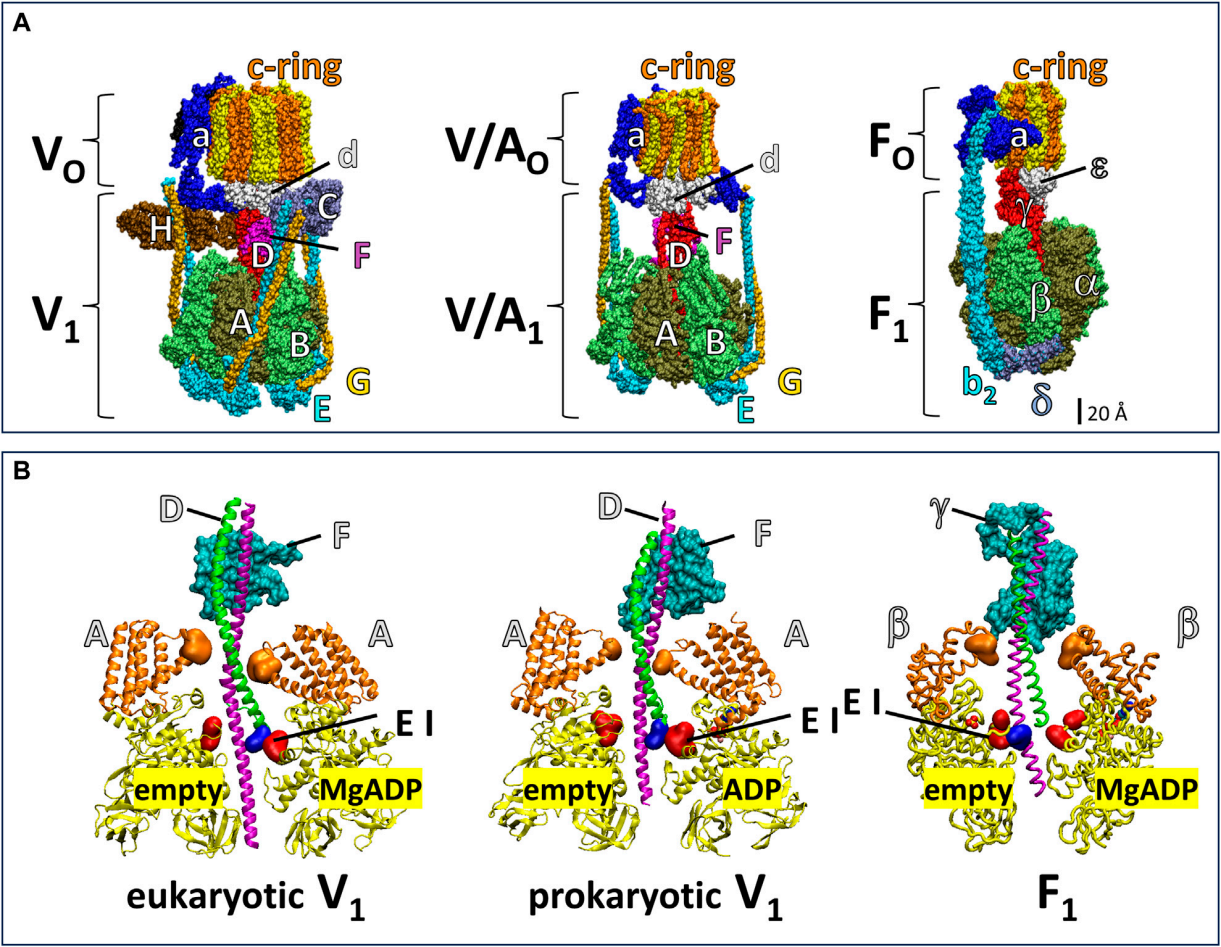
Subunit composition of eukaryotic V_1_V_O_ ATPase from *Saccharomyces cerevisiae* (PDB-ID 6O7W), V/A_1_A_O_ ATP synthase from *Thermus thermophilus* (PDB-ID 5GAR), and F_1_F_O_ ATP synthase from *E. coli* (PDB-ID 6OQR). **(A)** The rotors of V_1_ and V/A_1_ are composed of subunit D and subunit F. The former has an α-helical coiled-coil that extends through the core of the (AB)_3_-ring, while the latter globular protein helps dock subunit D to the V_O_ or V/A_O_ rotor c-ring. Analogous to subunits D and F, subunit γ serves as the F_1_ rotor, which contains a coiled-coil domain the extends through the F_1_ (αβ)_3_-ring and a globular domain that docks to the c-ring of F_O_. The eukaryotic V_1_V_O_ ATPase has unique regulatory subunits H and C and three peripheral stalks (EG)_3_ connecting V_1_ and V_O_, V/A_1_A_O_ ATP synthase has two peripheral stalks (EG)_2_, and F_1_F_O_ ATP synthase has one (b_2_) peripheral stalk. **(B)** Cross section of eukaryotic V_1_ from *Saccharomyces cerevisiae* (PDB-ID 7TMO), prokaryotic V_1_ from *E. hirae* (PDB-ID 5KNB); and F_1_ from *E. coli* (PDB-ID 3OAA). The helical coiled-coil of the rotors is comprised of the N-terminal helix (green) and C-terminal helix (pink) of V_1_ subunit F and F_1_ subunit γ and the globular foot of the rotor (cyan) is V_1_ subunit D and the globular domain of F_1_ subunit γ. Catalytic A subunits (V_1_) and β subunits (F_1_) have a helical domain (orange) and a Catch-Loop (red). The electrostatic interaction (EI) occurs between the rotor (blue) and the catch loop of the subunit-A about to release MgADP in V_1_, and between the rotor and the empty subunit β in F_1_.

The V-ATPase belongs to the super family of rotary ATPases (Muench et al., 2011; Schep et al., 2016; Sobti et al., 2020; Vasanthakumar et al., 2019) that also include the F-type, A-type and V/A-type (Figure 1). The eukaryotic V-ATPase is composed of the integral membrane V_O_ complex that provides the pathway for proton translocation, which is docked to the peripheral V_1_ complex (V_1_V_O_) (Benlekbir et al., 2012; Wang et al., 2020). The *Saccharomyces cerevisiae* V_O_ complex consists of subunits a, d, e, f in addition to ten proteolipids (subunits c, c’, c”), which form a ring structure (c-ring) (Mazhab-Jafari et al., 2016; Roh et al., 2018). The V_1_ complex is comprised of eight different subunits A_3_B_3_CDE_3_FG_3_H (Zhang et al., 2003; Zhang et al., 2008; Benlekbir et al., 2012). Alternating subunits A and B form a catalytic hexameric ring consisting of three AB heterodimers, each with a catalytic site to hydrolyze ATP (Arai et al., 2013). The V_1_ rotor (subunits D and F) is in the center of the (AB)_3_-ring and docks to the c-ring via V_O_ subunit d for catalytic coupling. Three peripheral stalks (E/G heterodimers) connect the V_1_-ring of three AB heterodimers to the V_O_-proton transfer domain. The peripheral stalks provide the docking site for the V_1_ regulatory H and C subunits in the V_1_V_O_ holocomplex (Sagermann et al., 2001; Drory et al., 2004; Diepholz et al., 2008; Oot et al., 2012). Together, the, EG_1-3_ heterodimers, subunit H, subunit C, and N-terminal domain of subunit a provide structural support between the ATPase and proton channel (Balakrishna et al., 2015).

The F_1_F_O_ and A_1_A_O_ complexes assemble into similar heterodimer rings that contain three catalytic sites surrounding a central rotor that is attached to a c-ring, which uses subunit a to facilitate proton translocation (Figure 1). However, the F-type and A-type motors have one and two peripheral stalks, respectively, and lack the eukaryotic V-type regulatory subunits H and C (Muench et al., 2011). The bacterial V/A-type ATP synthase, found in *Thermus thermophilus*, and bacterial V-type ATPase Na^+^ pump found in *Enterococcus hirae* contain two peripheral stalks and also lack the subunits H and C that are essential for regulation of eukaryotic V-types (Muench et al., 2011; Schep et al., 2016; Vasanthakumar et al., 2019; Sobti et al., 2020). While F-, A-, and V/A-type ATP synthases can synthesize and hydrolyze ATP, eukaryotic V-type ATPases are ATP hydrolysis-dependent proton pumps *in vivo* (Kane, 2006; Forgac, 2007), even though *Arabidopsis thaliana* V_1_V_O_ has been shown to synthesize ATP at exceptionally low ATP synthase rates *in vitro* (Hirata et al., 2000).

Eukaryotic V_1_ catalyzes ATP hydrolysis at the nucleotide-binding domains within the interface of the subunits A and B dimers (Arai et al., 2013; Wang et al., 2020), and most residues that facilitate ATP hydrolysis reside on V_1_ subunit A. Due to the different conformations of the three A subunits relative to subunit D of the rotor, these residues catalyze ATP hydrolysis via an alternating site mechanism (Kayalar et al., 1977; Boyer, 2002) where each catalytic site is in a different conformation (open/empty state, tight/substrate bound state, and loose/product release state) at any given time. ATP hydrolysis drives V-ATPase rotation of subunits DFd and the c-ring (Hirata et al., 2003). Bacterial *T. thermophilus* V/A_1_ was observed to rotate in an equivalent manner (Yokoyama et al., 2003). During rotational catalysis, carboxyl groups on the c-ring take up protons from the V_1_ side of the membrane via the input channel in V_O_ subunit a, which upon completing a full rotation, are deposited on the other side of the membrane via the subunit a output channel (Kawasaki-Nishi et al., 2001; Forgac, 2007; Oot et al., 2017). This enables V-ATPase-dependent proton pumping to maintain a non-equilibrium pH gradient across the cytoplasm and organellar membranes.

Eukaryotic V_1_V_O_ is regulated by a unique mechanism among the rotary ATPase superfamily, which results in the reversible dissociation of the V_1_-ATPase complex from the V_O_ proton-transfer complex (Kane, 1995; Sumner et al., 1995; Parra and Kane, 1998; Parra et al., 2014; Hayek et al., 2019). Reversible dissociation helps to maintain cellular pH homeostasis in coordination with the metabolic state of the cell (Parra and Kane, 1998; Hayek et al., 2019). Nutrient stress conditions such as limiting glucose induce ATP hydrolysis dependent dissociation of the V_1_ subunit C from V_1_V_O_, which in turn prompts dissociation of V_1_ from V_O_ and halts proton pumping until glucose is restored and the V_1_V_O_ holocomplex reassembles. Upon disassembly, a conformational change of V_1_ subunit H inhibits the futile hydrolysis of cytosolic ATP by the dissociated V_1_ that is uncoupled from proton transport (Parra et al., 2000; Oot et al., 2016; Oot et al., 2017). In this conformation, subunit H bridges the V_1_ rotor and a stator to halt rotation and trap an inhibitory Mg-ADP in one catalytic site of the autoinhibited V_1_. Glucose-dependent V-ATPase disassembly requires ATP hydrolysis (Parra and Kane, 1998), while reassembly involves the RAVE V-ATPase exclusive assembly factor (Jaskolka et al., 2021). The protein Oxr1p appears to aid in V-ATPase disassembly (Khan et al., 2022).

However, the mechanism of how rotational catalysis mediates disassembly and reassembly remains elusive. Structural studies have identified three states of *S. cerevisiae* V_1_V_O_ that differ by 120° rotational positions of the rotor relative to the asymmetric stator (Zhao et al., 2015). The rotary positions of these states are thought to correspond to the catalytic dwell positions of V_1_V_O_ when ATP hydrolysis occurs at one of the three catalytic sites. Only one of these rotary states appears to be optimal to initiate disassembly (Mazhab-Jafari et al., 2016; Oot et al., 2016). These structures captured snapshots of individual protein conformations, but single-molecule rotation studies are required to capture the nuances of the rotary mechanism.

Although progress has been made regarding rotational studies of bacterial V/A_1_-ATPase from *T. thermophilus* and V_1_-ATPase from *E. hirae* (Furuike et al., 2011; Iida et al., 2019), much less is known about the rotational mechanism of eukaryotic V_1_ rotation. Single molecules of *S. cerevisiae* V_1_V_O_ were observed to undergo ATPase-dependent counterclockwise rotation as viewed from the membrane (Hirata et al., 2003), which was in the same direction as has been observed for F_1_, A_1_, and V/A_1_ motors (Imamura et al., 2003; Spetzler et al., 2009; Furuike et al., 2011; Sielaff et al., 2016). In the *S. cerevisiae* V_1_V_O_ experiments, the c-ring was attached to the slide and rotation was monitored by an actin filament attached to subunit G (Hirata et al., 2003). However, the drag imposed by the actin filament and the limitation of measurement time resolution obscured other rotational details of this motor including changes in its angular velocity or whether dwells interrupt rotation. To date, only the F_1_-ATPase, A_1_-ATPase and bacterial V_1_-ATPase rotary motors have been characterized by single-molecule rotation studies under conditions that resolve dwells and angular velocity changes (Spetzler et al., 2009; Ishmukhametov et al., 2010; Sielaff et al., 2016; Ragunathan et al., 2017; Iida et al., 2019). The rotary positions of ATP binding and product release have been found to differ among the F_1_, A_1_, and bacterial V_1_ motors (Furuike et al., 2011; Martin et al., 2014; Suzuki et al., 2014; Sielaff et al., 2016; Martin et al., 2018; Iida et al., 2019; Kobayashi et al., 2020; Zarco-Zavala et al., 2020), although the mechanistic basis for these differences is a major unresolved question.

Årrhenius analysis of F_1_ rotation indicated that the first 60° of the 120° power stroke resulted from release of elastic energy, which was postulated to result from interactions between the rotor coiled-coil domain and the surrounding catalytic sites (Martin et al., 2018). The electrostatic interaction between highly conserved residues of the C-terminal helix of the rotor and catch loop residues (Greene and Frasch, 2003) of the empty catalytic site is thought to contribute significantly to the elastic energy that powers the first 60° of rotation. Notably, this electrostatic interaction in eukaryotic and prokaryotic V_1_-ATPases occurs between the N-terminus of the subunit F rotor and the catch loop of the catalytic site conformation that releases bound MgADP (Figure 1B).

Here, we characterized rotational dynamics of the eukaryotic *S. cerevisiae* V_1_-ATPase that lacks regulatory subunit H and subunit C using single-molecule studies with high resolution of time and rotational position. Rotation was observed in 120° power strokes separated by dwells comparable to the power strokes and catalytic dwells observed in F_1_, A_1_, and bacterial V_1_ and V/A_1_ ATPases. However, the power strokes were interrupted by dwells at rotary positions that occurred most frequently 45° and 112° after the end of the catalytic dwell. The results support a mechanism in which the 45° dwell results from dissociation of ADP while the 112° dwell late in the power stroke is the result of ATP binding to the empty site.

## Materials and methods

### Construction of a His-tagged V_1_ΔHC and site-directed mutagenesis

To generate *S. cerevisiae* that express the V_1_-ATPase for single molecule measurements, the regulatory subunits C and H were deleted, two cysteine substitution mutations (Y73C and T123C) were made on subunit D for biotinylation, and a 6xhis tag was added to c-terminus of subunit G for purification.

First, the genes encoding subunits C and H (*VMA5* and *VMA13*, respectively) in the *S. cerevisiae* strain SF838–5Aα genomic DNA were replaced with *NAT* and *KanMX* selectable marker genes through homologous recombination, respectively. Additionally, the subunit G gene (*VMA10*) was replaced with the *URA3* gene so the wild type subunit G would not compete for assembly in V_1_ because only the mutant containing the 6xhis tag was expressed. Mutant colonies were selected by inoculating the cells on SC + nourseothricin + kanamycin-uracil plates.

To generate V_1_ with a 6xhis tag on subunit G, yeast genomic DNA was isolated from SF838–5Aα cells. The subunit G gene (*VMA10*) was PCR amplified using primers that contained the His tag sequence, the PCR product was restriction digested, and the DNA fragment was subcloned the expression vector. Then, 5Aα *VMA5Δ::NAT, VMA10Δ::URA3, VMA13Δ::KanMX* cells were transformed with the recombinant plasmid. Mutant colonies were selected by inoculating the cells on SC-uracil-leucine plates.

To generate the cysteine substitution mutation for the biotinylation, the subunit D gene (*VMA8*) was PCR amplified from the genomic DNA, the PCR product was restriction digested, and the DNA fragment was subcloned into the pRS313 expression vector. The two cysteine substitution mutations (Y73C and T123C) were introduced through site-directed mutagenesis. This plasmid was used as a template to PCR amplify the mutant *VMA8* gene along with the *HIS3* gene from the vector using primers containing the 5′ and 3’ flanking regions of the *VMA8* gene. Then, the double cysteine mutant *VMA8-HIS3* PCR product was integrated into the genome of 5Aα *VMA5Δ::NAT, VMA10Δ::URA3, VMA13Δ::KanMX* cells containing pRS315-*VMA10-6His* through homologous recombination. Mutant colonies were selected by inoculating the cells on SC-uracil-leucine-histidine plates. Mutations were confirmed after each step with agarose gel electrophoresis and DNA sequencing.

### V_1_ΔHC purification

*Saccharomyces cerevisiae* 5Aα *VMA5Δ::NAT, VMA8-Y73C-T123C-HIS3, VMA10Δ::URA3, VMA13Δ::KanMX* - pRS315-*VMA10-6His* cells were grown in six 1L SC-histidine-leucine-uracil growth media at 30°C while shaking until the OD_600_ was on average 1.0 OD/mL. The cells were spun down at 5,000 rpm at room temperature for 5 min. The cell pellets were resuspended in 200 mL of spheroplast pretreatment buffer (100 mM Tris/HCl pH 9.4, 10 mM DTT) and spun down in the same condition. The cells were resuspended in 2% glucose solution and spun down. The cells were resuspended in spheroplast buffer (10 mM Tris/HCl pH 7.5, 1.2 M sorbitol, 40% glucose) to the final concentration of 15 OD/mL. Then, 1.5 U/μL zymolyase was added to the cell suspension to the final concentration of 1U/10 OD of cells. The cells were incubated at 30°C while shaking at 80 rpm for 60 min. After incubation, the spheroplasts were spun down at 3,000 rpm in 4°C for 5 min. The pellets were resuspended in spheroplast wash buffer and spun down in the same condition and the washing step was repeated two more times (spheroplast wash buffer: 6.8 mg/mL Yeast Nitrogen Base, 50 mM sodium phosphate dibasic, 50 mM succinic acid/NaOH pH 5.0, 2% glucose, 1.2 M sorbitol, 0.02 mg/mL histidine, 0.12 mg/mL leucine, 0.02 mg/mL adenine, 0.06 mg/mL lysine, 0.02 mg/mL arginine, 0.02 mg/mL tryptophan, 0.03 mg/mL tyrosine, 0.2 mg/mL threonine, 0.02 mg/mL methionine, 0.05 mg/mL phenylalanine, 0.02 mg/mL uracil). The following steps were done at 4°C. The cell pellet was homogenized in 20 mL of lysis buffer (PBS +1% (w/v) C_12_E_9_ 1mM PMSF, 5 μg/mL aprotinin, 2 μg/mL chymostatin, 1 μg/mL pepstatin A, 1 μg/mL leupeptin) and incubated in ice for 10 min. The samples were centrifuged at 30 k rpm (109,000 xg). After centrifugation, 10X binding buffer (0.5 M Tris/HCl pH 8.0, 1 M KCl, 400 mM imidazole, 50 mM MgCl_2_) was added to the supernatant to make the final concentration 1X. Finally, V_1_-ATPase was purified from the mixture by Ni-NTA chromatography, 1 mg of biotin maleimide was added to the column elution and incubated at 4°C for 15 min while shaking, and the sample was run through Sephadex G50 column equilibrated with storage buffer (50 mM Tris/HCl pH 8.0, 20 mM KCl, 2 mM ATP, 1 mM MgCl_2_, 15% glycerol). The purified, biotinylated V_1_-ATPase samples were aliquoted into 20 μL, quickly frozen, and stored at −80°C until use.

### ATP hydrolysis assay

The rate of ATP hydrolysis by the purified V_1_ΔHC was measured with an ATP-regenerating NADH-coupled assay (Lotscher et al., 1984). The measurement was made with the final concentration of 25 mM Tris/HCl (pH 8.0), 60 mM KCl, 2.5 mM phosphoenolpyruvate, 0.3 mM NADH, 17.5 Units pyruvate kinase (rabbit muscle, Sigma Aldrich), 25 Units L-lactate dehydrogenase (rabbit muscle, Sigma Aldrich), at varying concentrations of ATP including 1, 0.5, 0.2, 0.1, 0.05, 0.02, 0.01 mM, twice the MgCl_2_ concentration for each corresponding ATP concentration, and 3.22 × 10^−5^ mM of purified V_1_ΔHC (0.0174 mg/mL) in a final volume of 2.5 mL. The rate was determined in three replicates as the change in absorbance at 340 nm using a Cary 100 spectrophotometer with Peltier temperature control at 25°C. MgATP concentration was determined by the Maxchelator program MgATP calculator v1.3 using constants from NIST database #46 v8 (UC Davis Health).

### Single molecule gold nanorod rotation assay

The rotation of individual V_1_ΔHC molecules were observed with a single-molecule rotation assay using gold nanorods under a dark field microscope (Spetzler et al., 2009; Martin et al., 2014). Purified V_1_ΔHC molecules were immobilized on a microscope cover slip by the His-tag on the G-subunits, unbound molecules were washed off the slide with wash buffer (30 mM Tris/HCl pH 8.0, 10 mM KCl). The surface area of the cover slip that remained exposed around the bound V_1_ΔHC molecules was then coated with BSA-C, which prevented the gold nanorods from binding nonspecifically to the surface. The 80 × 40 nm gold nanorod (A12-50-600 purchased from Nanopartz) coated with Neutravidin was bound to the biotinylated subunit D, excess gold nanorods were washed off with the wash buffer, and rotation buffer (1 mM MgCl_2_, 2 mM ATP, 30 mM Tris/HCl pH 8.0, 10 mM KCl) was added to the cover slip (Spetzler et al., 2009). The rotations of individual molecules were observed by measuring the fluctuation of polarized red light scattered off the AuNR using a single-photon detector. In each molecule observed, the orientation of the polarizing filter was adjusted to align with the minimum light intensity position that corresponded to one of the three catalytic dwells. The sinusoidal fluctuation of the polarized red-light intensity was measured as the gold nanorod rotated from 0° to 90° relative to the catalytic dwell position. Measurements were taken in the form of 5 s datasets at a frame rate of 100 kHz. The standard error measurements of histograms of the intensity of red light scattered from a single nonrotating nanorod fixed to a slide as a function of the rotational position of the polarizer varies between about 0.02 and 0.12° as the scattered light intensity varied between minimum and maximum values (Ishmukhametov et al., 2010).

## Results

A strain of *S. cerevisiae* lacking the genes for the V_1_ regulatory subunit H and subunit C was used to express the V_1_-ATPase complex (hereafter V_1_ΔHC). V_1_ΔHC resembles the V_1_ naturally found in the cytosol, which lacks subunit C. However, unlike the cytosolic V_1_, V_1_ΔHC is not inhibited by subunit H. For single-molecule rotation studies, subunit G of V_1_ΔHC was genetically modified to add a 6xhis tag to the C-terminus, and subunit D mutations Y73C and T123C were made to enable covalent modification by biotin maleimide. The V_1_ΔHC construct exhibited ensemble ATP hydrolysis commensurate with V-ATPases and single molecule rotational ATPase activity consistent with the general mechanism of the rotary ATPases. However, V_1_ΔHC rotation differed in notable ways.

### Ensemble ATPase assays

**FIGURE 2:**
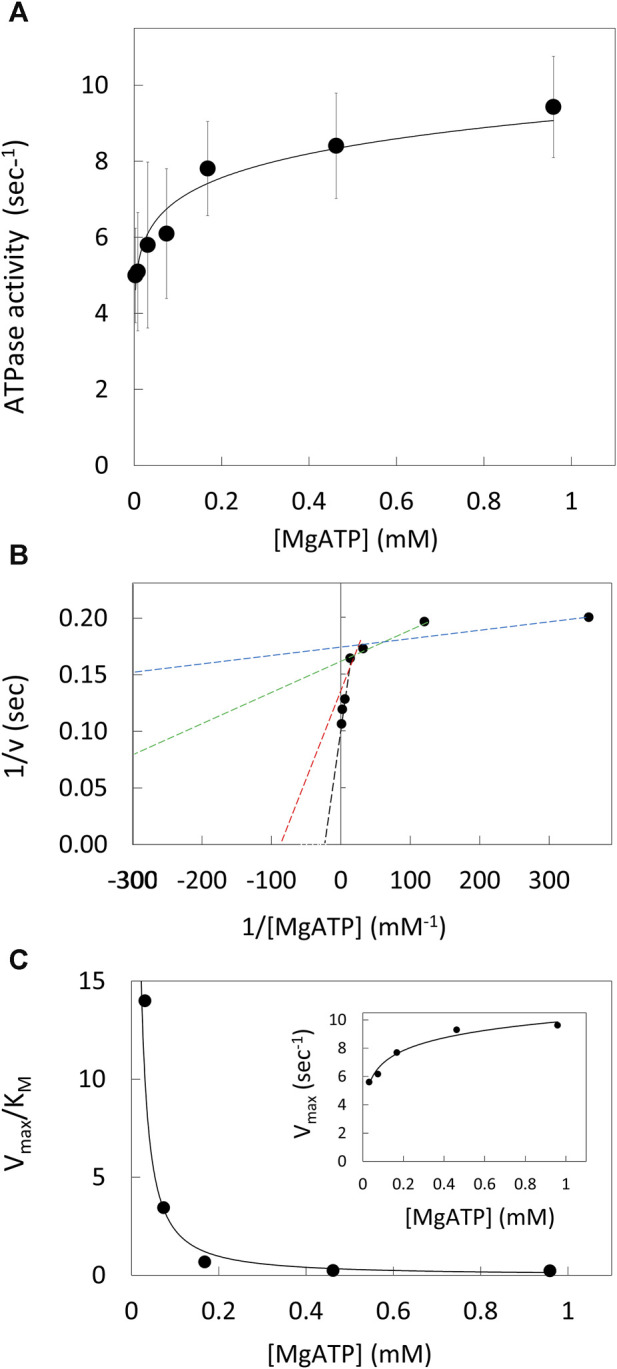
V_1_ΔHC ATPase Ensemble Assays. **(A)** Rate of ATP hydrolysis vs. MgATP concentration at 25°. **(B)** Double reciprocal plot of ATP hydrolysis rates. Values of K_M_ and V_max_ were determined from the intersects on the *x* and *y*-axes (−1/K_M_ and 1/v), respectively, of a straight line of each three consecutive MgATP concentrations. **(C)** Values of V_max_/K_M_ vs. [MgATP]. Inset: V_max_ values vs. [MgATP].

The ATPase activity of purified V_1_ΔHC *versus* the MgATP concentration (Figure 2A) was measured using an ensemble coupled assay with pyruvate kinase and lactic dehydrogenase at 25°C. The apparent V_max_ was observed at 990 μM MgATP. The double-reciprocal plot of ATPase activity versus [MgATP] was not linear (Figure 2B). As such, V_1_ΔHC did not exhibit simple Michaelis-Menten kinetics, which would have a linear dependence in a double reciprocal plot defined by Eq. 1,

$$1/v=\left(K_{M}/V_{\max}\right)\left(1/\left[\text{MgATP}\right]\right)+1/V_{\max}$$ (1)

where v is the observed velocity at a given MgATP concentration, V_max_ is the apparent maximum velocity and K_M_ is the Michaelis constant. The kinetic values were determined from Eq. 1 for each of three consecutive ATP concentrations in the double-reciprocal plot. The V_max_ increased with MgATP concentration to a maximum turnover number of 9.6 s^−1^ (Figure 2C, inset). The time required to hydrolyze an ATP at 990 μM MgATP (saturating) as well as at 490 μM and 5.7 μM MgATP, which were rate-limiting, is shown in Table 1. The V_max_/K_M_ values decreased 30-fold *versus* MgATP in a non-linear manner (Figure 2C), which indicates that the affinity of the empty catalytic site to bind MgATP decreases as V_1_ΔHC approaches saturating MgATP concentrations. At saturating MgATP concentrations, the K_M_ was 41.7 μM. Changes in both V_max_ (∼2-fold) and K_M_ (100-fold) contributed to the decrease in V_max_/K_M_ *versus* MgATP.

**TABLE 1:** Durations of V_1_ΔHC power strokes, dwells and ATP consumption derived from single-molecule measurements compared to those derived from the ensemble ATPase assay *versus* MgATP concentration.

	Single-molecule rotation measurements					Ensemble ATPase assays	
[MgATP] (μM)	Number of power strokes	Number of V_1_ΔHC^a^	Average power stroke duration (μs)	Average catalytic dwell duration (ms)	Average ms/ATP^b^	ms/ATP^c^	% V_1_ΔHC active^d^
990	10,274	48	625	7.2	7.8	105.2	7.4
490	1,024	8	745	12.3	13.0	118.9	11
5.7	1,625	14	910	13.4	14.3	200.0	7.2

^a^ Number of V_1_ΔHC, molecules examined.

^b^ Average ATP, consumption time (sum of power stroke and catalytic dwell durations).

^c^ Average ensemble ATP, consumption time (1/apparent V_max_).

^d^ Percent of V_1_ΔHC, molecules actively consuming ATP, in ensemble assay (single-molecule ms/ATP/ensemble ms/ATP).

### Single molecule rotation assays

**FIGURE 3:**
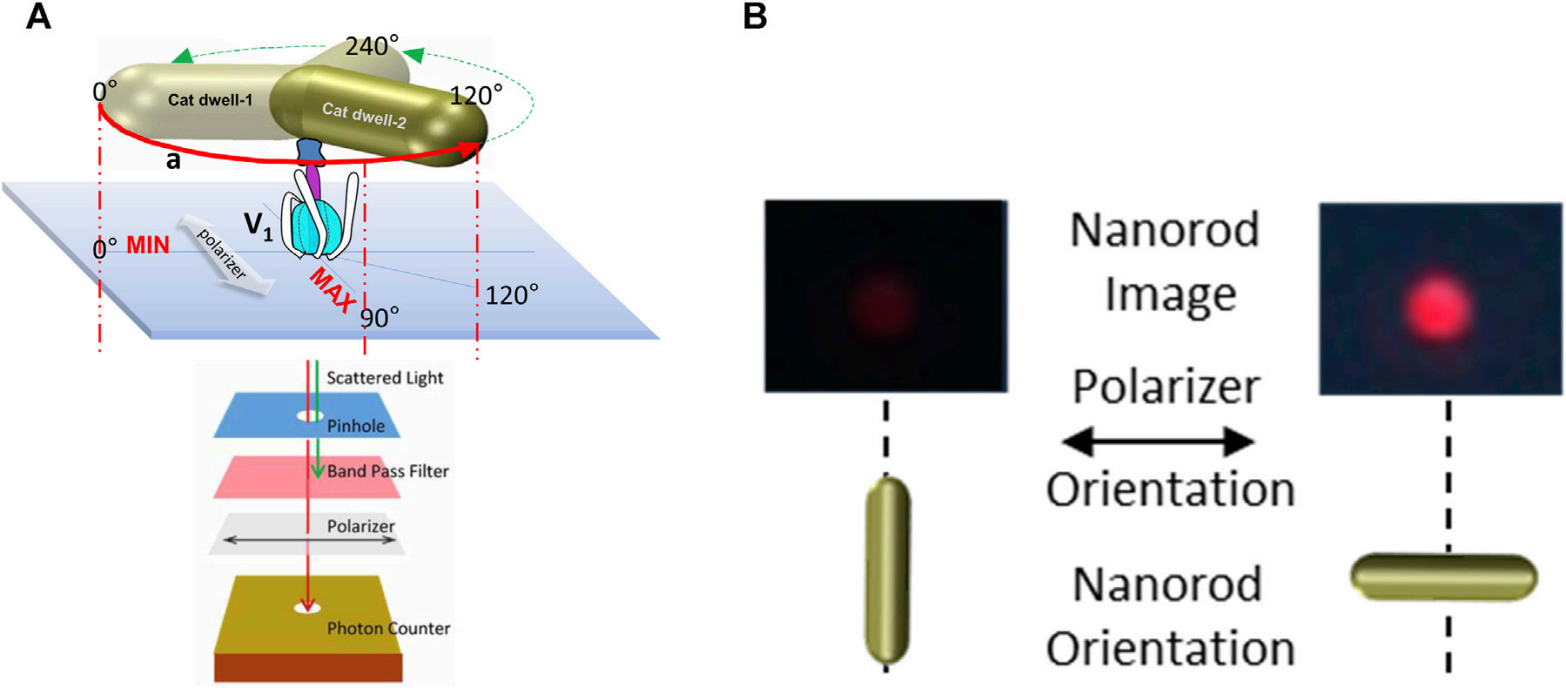
Use of red light scattered from a 75 × 35 nm gold nanorod (AuNR) for single-molecule rotation measurements. **(A)** Molecules of V_1_ΔHC were attached to a cover slip via His-tags on subunit E, and an AuNR was attached to each V_1_ΔHC via biotinylated cysteines on subunit D created by mutagenesis (Y73C and T123C). Red scattered light was recorded by an avalanche photo diode after passing through a pinhole to allow light from a single AuNR, a polarizer, and a band-pass filter to remove shorter wavelengths of light. **(B)** Intensity of red light from a single AuNR when the short and long axes of the AuNR are parallel to the direction of the polarizer.

To measure ATP hydrolysis-dependent rotation of subunit D, purified V_1_ΔHC molecules were immobilized on a microscope cover slip by the His-tags on the three G subunits, and a 35 × 75 nm NeutrAvidin-coated gold nanorod (AuNR) was attached to the biotinylated Y73C and T123C mutations of subunit D (Figure 3A). Rotation of single V_1_ΔHC molecules was observed in the presence of MgATP by the changes in intensity of polarized red light that scatters from the AuNR. While concentrations of the MgATP complex used in ensemble ATPase measurements and single-molecule experiments were the same, the Mg:ATP ratio was 2:1 and 1:2, respectively. The 1:2 ratio used in the single-molecule experiments was intentional to minimize MgADP inhibition. These measurements were also carried out in the absence of an ATP regenerating system because the small number of V_1_ molecules on the cover slip did not consume significant amounts of ATP during the assay. After the scattered red light passed through a high wavelength band-pass filter to remove light of wavelengths shorter than 600 nm, and a polarizing filter that could be rotated to specific orientations, the light intensity changes were quantified by an avalanche photodiode with a 50 ns time resolution. In this manner the intensity of scattered red light was at a minimum and maximum when the polarizer was oriented perpendicular and parallel to the long axis of the AuNR (Figure 3B).

**FIGURE 4:**
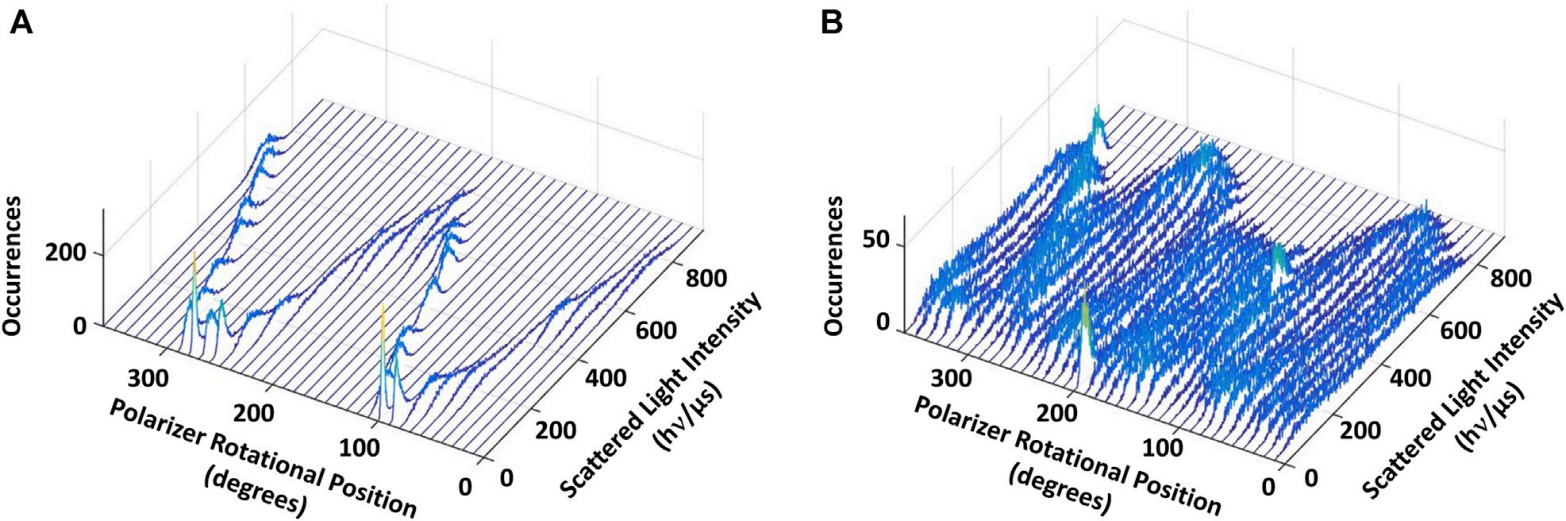
Eukaryotic V_1_ΔHC catalyzes ATPase-dependent 120° power strokes that occur on a sub-ms time scale that are separated by ms-duration dwells. **(A)** Consecutive histograms of light intensity scattered from a AuNR attached to a molecule of V_1_ΔHC that was not rotating show a single sinusoidal dependence. **(B)** Consecutive histograms of light scattered from a AuNR attached to a molecule of V_1_ΔHC catalyzing ATP-dependent rotation at saturating MgATP concentration show three overlapping sinusoidal intensities off-set by 120°. Light intensity was collected from the AuNR at 1 kHz (data collected at 1 ms intervals) after each 10° rotation of the polarizer.

Figure 4 shows the results of a polarizer rotation measurement when the AuNR is attached to subunit D of a single V_1_ΔHC molecule. In this experiment, the light intensity scattered from the polarizer was rotated 10° in successive 5 s intervals for a total of 360°. During this time, the intensity of light scattered from the AuNR was acquired by the single-photon counter at 1 kHz (equivalent to 1,000 fps). Since red light scatters specifically from the long axis of the AuNR, the scattered light intensity when subunit D was not rotating in this experiment (Figure 4A, control) varied in a sinusoidal manner relative to the rotational axis of the polarizer (Spetzler et al., 2006; Ishmukhametov et al., 2010; Frasch et al., 2022).

In the presence of 990 μM (saturating) MgATP (Figure 4B), three sinusoidal intensity curves were observed in the polarizer rotation measurement, which were off-set from each other by 120°. The 1 kHz data acquisition rate provides a time resolution of 1 ms such that the light intensities reported the rotational position of the AuNR primarily when it was in the same position for more than 1 ms. This indicates that, in the presence of saturating MgATP, subunit D stopped at three rotary positions separated by 120° that each lasted >1 ms. To do so, subunit D rotated between these dwells with a power stroke that occurred too fast for the 1 kHz data acquisition rate to record as much scattered light as was observed during the dwells. These dwells are hereafter referred to as catalytic dwells, since they are comparable to the duration and rotary positions of catalytic dwells observed between the 120° ATPase-driven power strokes of F_1_, A_1_, V/A_1_ and bacterial V_1_ ATPases (Spetzler et al., 2006; Furuike et al., 2011; Minagawa et al., 2013; Sielaff et al., 2016; Zarco-Zavala et al., 2020).

To resolve the intermediate positions of the 120° rotational events between the dwells observed in Figure 4, the intensity of light scattered from the AuNR was sampled at 200 kHz (5 μs per data point). Prior to the 5 s data acquisition of each V_1_ΔHC molecule, the rotational position of the polarizer was set so that the scattered light intensity was at a minimum at one of the three catalytic dwell positions. As a result, the light intensity of the subsequent power stroke increased from a minimum through a maximum as the AuNR rotated from 0° to 90° relative to that catalytic dwell, then decreased until it reached the next catalytic dwell upon rotating 120°. An arcsine^1/2^ function of light intensity (Sielaff et al., 2016; Martin et al., 2018) was used to calculate rotational position *versus* time. Changes in rotary position *versus* time (angular velocity) for each power stroke were then averaged and binned to every 3° of rotation, where 0° and 120° refer to catalytic dwell positions.

**FIGURE 5:**
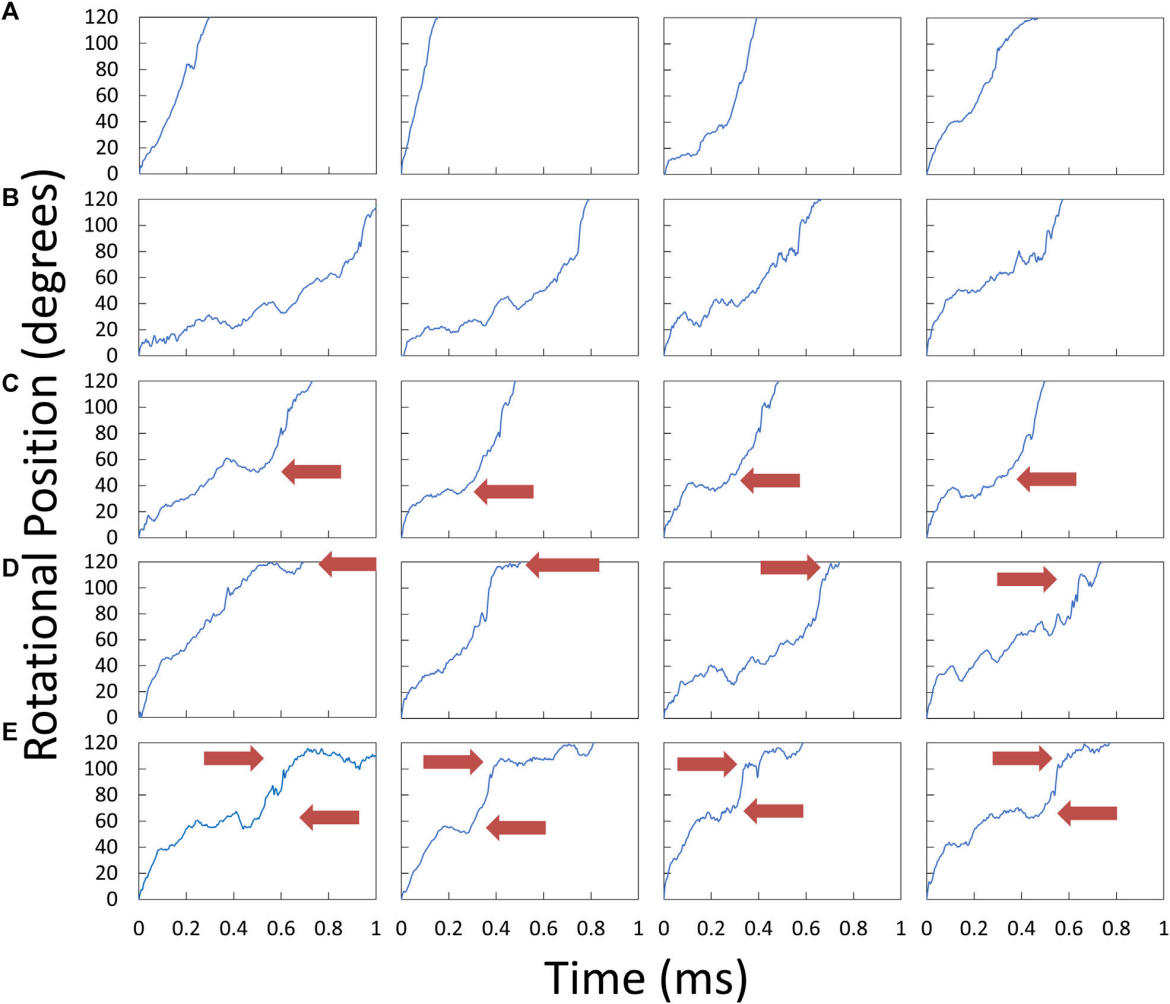
Examples of V_1_ΔHC power strokes plotted as rotary position vs. time. Scattered red light intensity from the AuNR was collected at 200 kHz from power strokes that began at the minimum light intensity at the end of a catalytic dwell (0°) and passed through the maximum light intensity (90°). Red arrows indicate the position at which power stroke rotation was clearly interrupted by a dwell. **(A)** Power strokes that rotated continually for 120°. **(B)** Power strokes that rotated in a faltering manner with small oscillations. **(C)** Power strokes interrupted midway by a dwell. **(D)** Power strokes interrupted by a dwell near the end. **(E)** Power strokes with dwells midway and near the end.

Examples of V_1_ΔHC power strokes that were used to determine the angular velocity profiles *versus* rotary position are shown in Figure 5. Many V_1_ΔHC power strokes rotated continuously (Figure 5A), which are comparable to *E. coli* F_1_ power strokes at saturating MgATP (Martin et al., 2018). However, many other V_1_ΔHC power strokes rotated in a faltering manner (Figure 5B) with frequent small oscillations. Some contained a clearly defined dwell during the first 60° of the power strokes (Figure 5C) even in the presence of saturating MgATP, while other power strokes contained a dwell near the end of the power stroke (Figure 5D). Although the power strokes shown were observed in the presence of 5.7 μM MgATP, all four types were present at all three of the MgATP concentrations examined.

**FIGURE 6:**
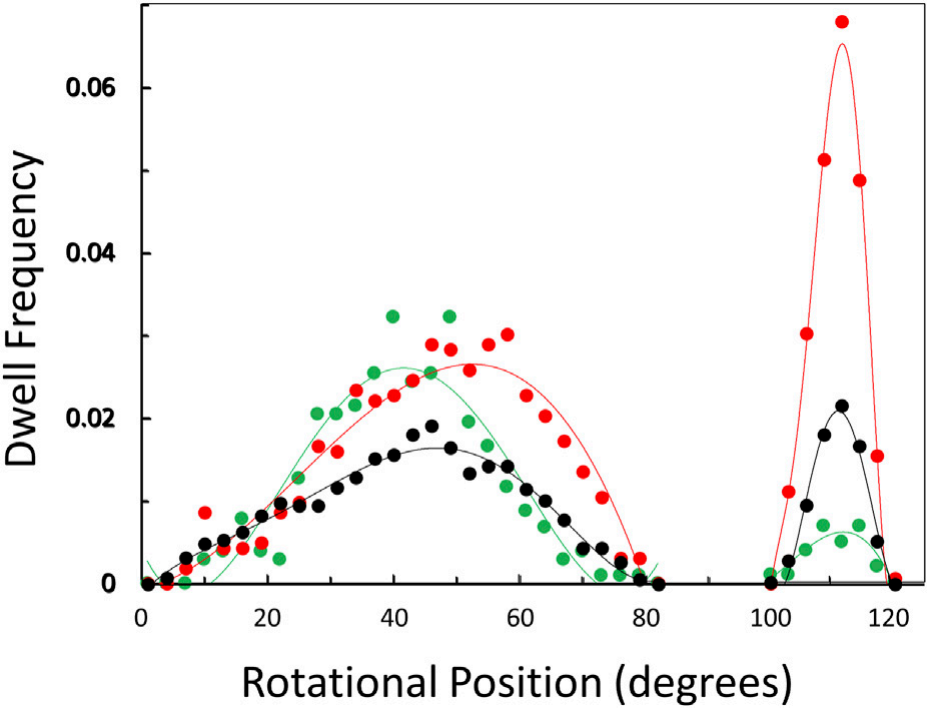
Observation of dwells occurring maximally at ∼45° and 112° during a power stroke *versus* MgATP concentration. Distribution of rotational positions of dwells observed at 990 μM (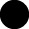), 490 μM (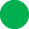), and 5.7 μM (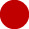) MgATP. The frequency that the dwell occurred at a rotational position is shown for the subset of power strokes that contained a dwell that interrupted the power stroke.

The distribution of rotary positions during the power stroke when V_1_ΔHC dwells occurred (Figure 6) shows that dwells were most commonly observed ∼45° and 112° after the catalytic dwell. The distribution of the former dwell was significantly broader than the latter, and the peak of the distribution observed at 990, 490, and 5.7 μM MgATP appeared to occur at 45°, 50°, and 40°, respectively (hereafter designated the 45° dwell). This is the approximate rotary position at which F_1_-ATPases gives rise to an “ATP-binding” dwell when MgATP is rate-limiting (Yasuda et al., 2001; Bilyard et al., 2013; Martin et al., 2014; Sobti et al., 2021).

The percentage of power strokes examined that contained a 45° dwell increased from 24% at saturating MgATP by 6% and by a further 8% at the rate-limiting MgATP concentrations of 490 and 5.7 μM MgATP, respectively (Table 2). The 184 ± 2 μs average duration of these dwells did not change significantly at the rate-limiting MgATP concentrations. However, the occurrence of the 112° dwell increased by ∼3-fold at 5.7 μM MgATP with respect to that of the 8.5% occurrence observed at saturating MgATP. The duration of these dwells increased by 21% (391 μs) at 5.7 μM MgATP.

**TABLE 2:** Occurrence and duration of dwells that interrupt the V_1_ΔHC power stroke *versus* MgATP concentration.

[MgATP]	45° dwell		112° dwell	
(μM)	% occurence^a^	Average duration (μs)	% occurence^a^	Average duration (μs)
990	24.0	184 ± 2	8.5	322 ± 5
490	30.2	183 ± 6	3.4	265 ± 25
5.7	38.9	194 ± 5	23.6	391 ± 10

^a^ Percent occurrence of dwell *versus* total power strokes analyzed at each MgATP, concentration.

**FIGURE 7:**
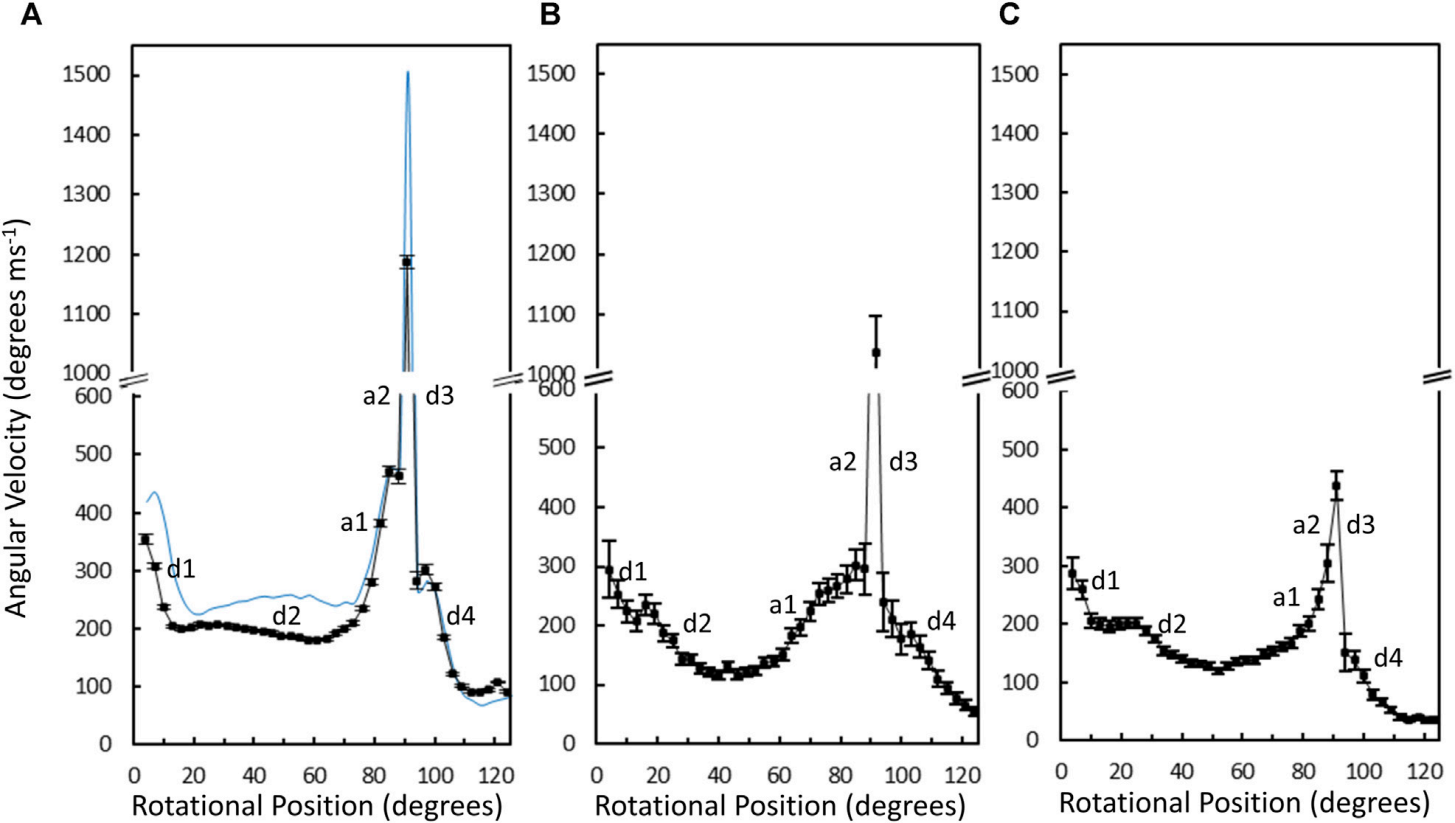
Average angular velocity vs. rotational position of V_1_ΔHC (black) *versus E. coli* F_1_ (blue) power strokes at 990 μM Mg-ATP **(A)**, 490 μM MgATP **(B)**, and 5.7 μM MgATP **(C)**. Rotational positions of decelerations (d) and accelerations (a) are numbered in the order that they occur during the power stroke.

The average angular velocity of the V_1_ΔHC power stroke vs. rotary position in the presence of saturating (990 μM) MgATP was calculated from the data of 10,274 power strokes examined from 48 V_1_ΔHC molecules (Figure 7A). The initial average velocity as the catalytic dwell ended was ∼350°∙ms^–1^. The V_1_ΔHC decelerated to ∼200°∙ms^–1^ at ∼13° (d1-deceleration), then slowly decelerated further from ∼23° to ∼180°∙m^–1^ at 60° (d2-deceleration). The rate subsequently accelerated to ∼470°∙ms^–1^ at 85° (a1-acceleration), and then rapidly accelerated to briefly reach a rate of ∼1,200°∙ms^–1^ at ∼90° (a2-acceleration) before decelerating at ∼93° (d3-deceleration). The rate returned to 300°∙ms^–1^ at ∼100° then decelerated to 100°∙m^–1^ (d4-deceleration) as it approached the next catalytic dwell at 120°. The V_1_ΔHC angular velocity profile was closely similar to that of the *E. coli* F_1_-ATPase (Martin et al., 2018) with the exception that the angular velocity of the latter was significantly slower during the first 70°, and was 21% slower during the spike in velocity at 90° (Figure 7A).

Changes in the angular velocity profile of the V_1_ΔHC power stroke vs. rotary position were observed when measured at 490 μM MgATP and at 5.7 μM mgATP (Figures 7B, C), which gave rise to ATPase rates that were 86% and 65% of that observed at 990 μM MgATP, respectively. In the presence of 490 μM MgATP (Figure 7B), the rates after the d2-deceleration, after the a1-acceleration, and after the d3-deceleration were ∼100°∙ ms^–1^ at ∼45°, ∼200°∙ ms^–1^ at 85°, and ∼180°∙ ms^–1^ at 100°. These rates corresponded to rate decreases of 2-fold, 2.4-fold, and 1.7-fold, respectively, from those observed at saturating MgATP. When measured at 5.7 μM MgATP (Figure 7C), the average V_1_ΔHC angular velocity profile showed additional decreases in velocity from that observed 490 μM MgATP between rotary positions 60° and 120°. Notably, the 5.7 μM MgATP velocities at 85°, 90°, and 100° were 230°∙ ms^–1^, 440°∙ ms^–1^, and 110°∙ ms^–1^, respectively, which represents decreases of 2.0-fold, 2.7-fold, and 2.7-fold from that observed at saturating MgATP.

The average time required for power strokes to rotate between catalytic dwells were calculated from the angular velocity profiles at each MgATP concentration (Table 1), since each profile reports the time required for each three degrees of rotation. At saturating MgATP (990 μM MgATP), the average power stroke duration was 625 μs while the average power stroke durations at the rate-limiting MgATP concentrations of 490 μM, and 5.7 μM MgATP were 745 and 910 μs, respectively. The power stroke durations at these limiting MgATP concentrations were 1.19-fold and 1.46-fold longer than that measured at saturating MgATP. These increases were comparable to the 1.16-fold and 1.53-fold longer times required to consume an ATP as determined from the ensemble ATPase measurements in the presence of 490 μM and 5.7 μM MgATP, respectively (Table 1). These results support the conclusion that the additional time required for MgATP to bind to the empty catalytic site when MgATP is rate-limiting is evident as a decrease in angular velocity during the power stroke.

**FIGURE 8:**
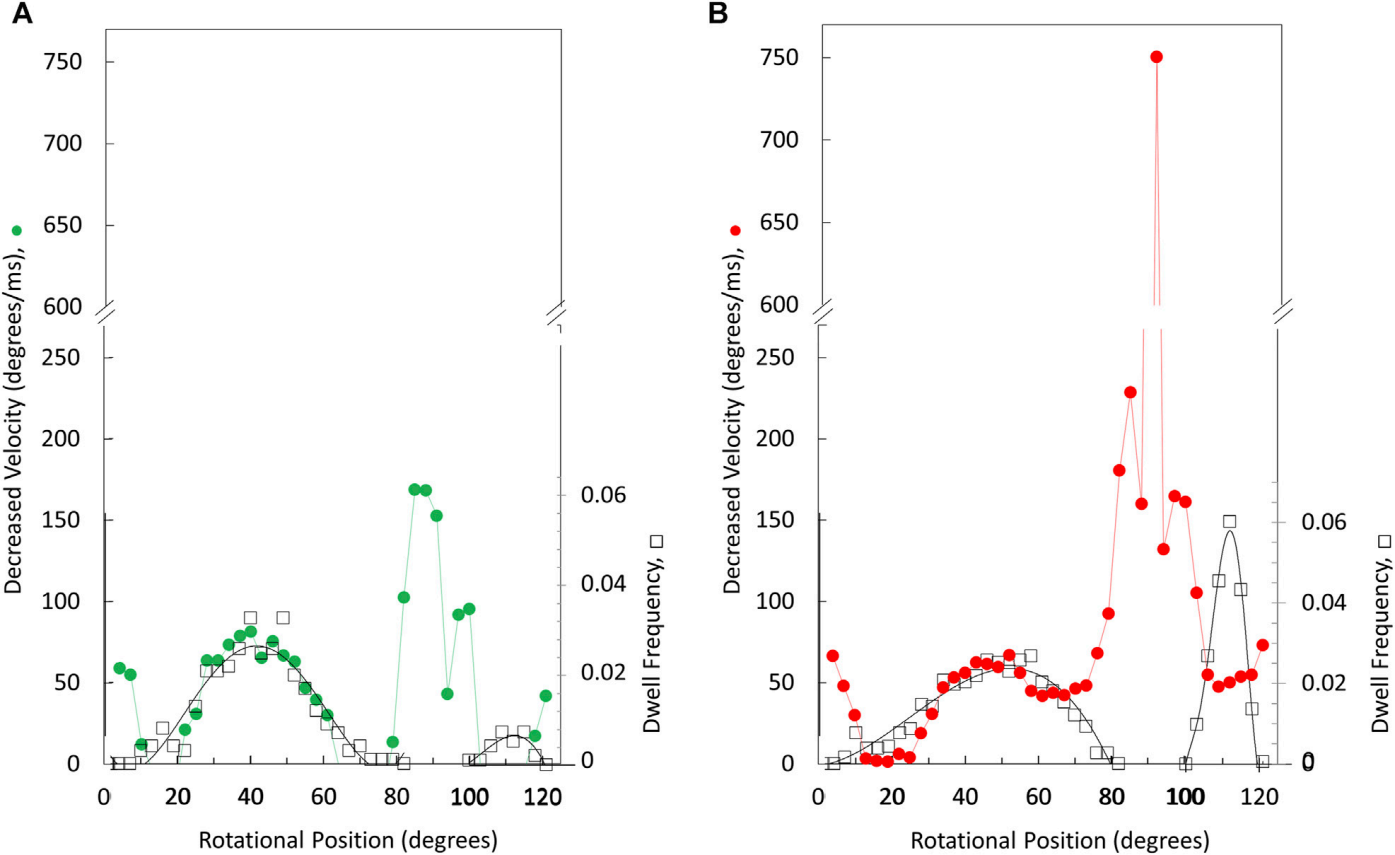
Rotary positions where decreases in average angular velocity occur at limiting *versus* saturating MgATP. Extent of rate decreases between 490 and 990 μM MgATP **(A)** and between 5.7 μM *versus* vs. 990 μM MgATP **(B)**. Distribution of the dwell occurrence that interrupts the power stroke (black squares) at 490 μM MgATP **(A)** and at 5.7 μM MgATP **(B)** where the scale of dwell frequency was normalized to the decreases in angular velocity of the 45° dwell.

The angular velocity profiles (Figure 8) were determined from the average of several thousand power strokes. Consequently, the decreases in average angular velocities observed at limiting MgATP may be due to slower rotation or may occur if a proportion of the power strokes briefly stop rotating, which would be observed as a dwell. The extent that limiting MgATP caused decreases in the angular velocity *versus* rotary position was determined by taking the difference angular velocity profiles at limiting MgATP from that at saturating MgATP (Figures 8A, B). When compared to the distribution of dwells (black squares) there is a clear correlation between the incidence of dwells and the decreases in angular velocity of the power stroke, but the frequency did not change as MgATP became increasingly limited. This suggests that the 45° dwell does not result from MgATP binding. However, the largest decreases in power stroke angular velocity when MgATP is rate-limiting was observed between 80° and 100° when dwells did not occur. Consequently, these results indicate that the major contribution to the decrease in ATPase rate when MgATP is limiting occurs between 80° and 120°.

The consumption of each ATP requires a consecutive catalytic dwell and power stroke. The power strokes analyzed here are one of three required for a complete rotation of subunit D. Based on the number of power strokes analyzed for each V_1_ΔHC molecule during the 5 s data acquisition period, and the average power stroke durations obtained directly from the angular velocity profiles, the average duration of catalytic dwells was determined by subtraction of the time consumed by the power stroke from the total data acquisition time (Table 1). The average catalytic dwell durations in the presence of 990 μM, 490 μM, and 5.7 μM MgATP were calculated to be 7.2 ms, 12.3 ms, and 13.4 ms, respectively. The average durations of the power strokes and catalytic dwells were consistent with the polarizer rotation results that have a minimum time resolution of 1 ms (Figure 4).

The average time required to consume an ATP molecule was also calculated from the sum of the average durations of the power stroke and the catalytic dwells (Table 1). In the presence of 990, 490, and 5.7 μM MgATP, the average times to consume an ATP were calculated from the single-molecule data to be 7.8, 13.0, and 14.3 ms, respectively. It is noteworthy that the times required to consume ATP as measured by the ensemble ATPase assay (Figure 2) at 990, 490, and 5.7 μM MgATP were 105.2, 118.9, and 200 ms (Table 1). These times were considerably longer than those determined by other single-molecule studies, where each molecule was known to be undergoing ATPase-dependent rotation. The ensemble assays reported the average of many V_1_ΔHC molecules without knowing how many molecules are actively consuming ATP. By comparing the ensemble and single-molecule results, we estimate that 7%–11% of the V_1_ΔHC molecules were actively consuming ATP at any moment in the ensemble assay (Table 1).

## Discussion

**FIGURE 9:**
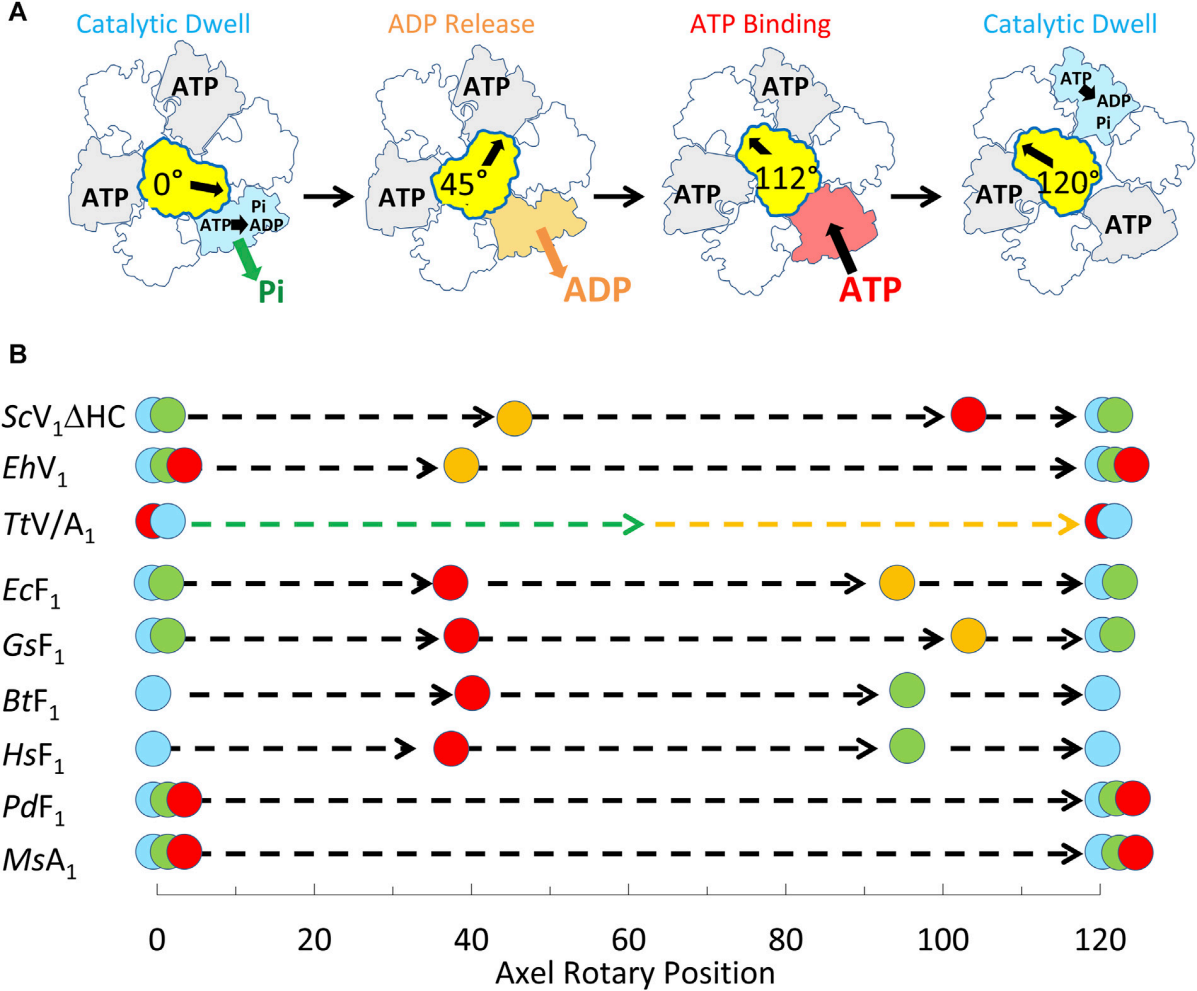
Mechanism of V_1_ΔHC relative to other members of the rotary ATPase super-family. **(A)** Eukaryotic V_1_-ATPase rotational mechanism. Rotary positions of *Saccharomyces cerevisiae* V_1_ΔHC MgATP binding and MgADP release based on results presented here. **(B)** Comparison of the rotational positions of events of eukaryotic V_1_ΔHC to other rotary ATPases. Mechanistic events are shown that occur relative to the catalytic dwell (0° and 120°) including ATP binding (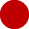), ATP hydrolysis (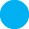), ADP dissociation (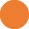), and Pi release (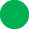). Species listed include *Saccharomyces cerevisiae* (*Sc*V_1_ΔHC), *Enterococcus hirae* (*Eh*V_1_) (Iida et al., 2019), *Thermus thermophilus* (*Tt*V/A1) (Furuike et al., 2011), *Escherichia coli* (*Ec*F1) (Martin et al., 2014; Martin et al., 2018); *Geobacillus stearothermophilus* (*Gs*F1) (Sielaff et al., 2016); *Bos taurus* (*Bt*F1) (Kobayashi et al., 2020); *Homo sapiens* (*Hs*F1) (Suzuki et al., 2014); *Paracoccus denitrificans* (*Pd*F1) (Zarco-Zavala et al., 2020); *Methanosarcina mazei* (*Ms*A1) (Sielaff et al., 2016).

The single-molecule results of eukaryotic V_1_ΔHC ATPase-dependent rotation presented here are consistent with a mechanism in which subunits D and F rotate in 120° power strokes separated by catalytic dwells when ATP hydrolysis occurs. This is supported by the presence of 120° power strokes that last for 0.63 ms–0.91 ms separated by longer 7.2–13.4 ms duration dwells. These power strokes have the same velocity profile as those of the F_1_-ATPases and A_1_-ATPases examined except for the magnitudes of the velocities (Sielaff et al., 2016; Ragunathan et al., 2017; Martin et al., 2018). These longer dwells are also consistent with catalytic dwells observed in F_1_-ATPases. These mechanistic features are shared by all members of the super family of rotary ATPases examined to date (Figure 9) even though these rotary ATPases have been found to vary in the rotational positions where ATP binds, as well as where ADP and Pi are released. The results presented here support the mechanism of the V_1_ΔHC shown in Figure 9A, and provide important new insight concerning the molecular basis for the differences in rotary positions of substrate binding and product release among these rotary motors.

The V_1_ΔHC rotation differed in notable ways from the more thoroughly studied F_1-_ATPase. First, rotation during the V_1_ΔHC power strokes often faltered with small back and forth oscillations during the first 60° of rotation subsequent to the catalytic dwell. Second, the V_1_ΔHC power strokes often contained a dwell occurring 40°–50° and/or a dwell at 112° after the catalytic dwell (8° before the subsequent catalytic dwell). Third, as the MgATP concentration became increasingly limited, the duration of the power stroke lengthened not only as the result of an increase in the occurrence and/or duration of 45° and 112° dwells, but also because the angular velocity decreased between 80° and 100° when dwells did not occur (Figure 8).

Interactions between the rotor and the catalytic sites provide clues to the differences in V_1_ΔHC rotation (observed here) from that of F_1_ ATPases. In both ATPases, the rotors are surrounded by contacts at the tip of the C-terminal helical domains (CHDs) of each subunit in the (AB)_3_-ring and by the (αβ)_3_-ring, where the catalytic sites are primarily located on the A-subunits and β-subunits. Both V_1_ and F_1_ have a strong electrostatic interaction between highly conserved catch-loop residues from one of the three catalytic sites with their respective rotors. These residues are A/D422, A/S424, and A/D425 and βD301, βT304, and βD305 in *S. cerevisiae* V_1_, and *E. coli* F_1_, respectively. However, the location of the rotor residues that form the electrostatic interaction differs significantly. In *S. cerevisiae* V_1_-ATPase, these residues (D/R12) are on the shorter N-terminal helix of the subunit D coiled-coil (Vasanthakumar et al., 2022) while these F_1_ residues (γQ269 and γR268 in *E. coli*) are on the longer C-terminal helix of the coiled-coil. As a result, V_1_ subunit D forms electrostatic interactions with the catch-loop of the subunit A catalytic site that is about to release ADP (loose conformation), while F_1_ subunit γ interacts electrostatically with the catch-loop of the empty β subunit (open conformation). Mutations of any of the residues that comprise this electrostatic interaction in *E. coli* F_1_ and *S. cerevisiae* V_1_-ATPase result in dramatic losses of catalytic activity (Greene and Frasch, 2003; Boltz and Frasch, 2006; Arsenieva et al., 2010).

Elastic coupling powered by ATP hydrolysis provides an explanation for faltering rotation (throughout first 60°) during *S. cerevisiae* V_1_ΔHC power strokes, which result from small oscillations and the occurrence of 45° dwells even at saturating MgATP. In F_1_, the restraints on the rotor imposed by the surrounding CTHs and by the catch loop interactions impose elastic strain by twisting the coiled-coil of the rotor. Unwinding this coiled-coil spring then powers the first 60° of rotation (Martin et al., 2018; Frasch et al., 2022). If the first 60° of V_1_ rotation is also powered by unwinding the coiled-coil of the rotor, the spring constant is likely to be decreased due to the subunit D short helix location and its electrostatic interaction to the ADP release catalytic site.

The V_1_ subunit D electrostatic link to the catalytic subunit conformation that releases ADP also suggests that the 45° dwells result from the dissociation of ADP. *E. hirae* V-ATPase has equivalent subunit D residues forming the electrostatic interaction with the ADP-release conformation (Suzuki et al., 2016). Of the rotary ATPases studied to date (Figure 9B), the *E. hirae* V-ATPase appears most closely related to eukaryotic V-ATPases. *E. hirae* V_1_V_O_ is an ATPase-dependent Na^+^ pump that is incapable of ATP synthesis. Single-molecule studies of purified *E. hirae* V_1_ show the presence of a dwell that occurs 40° after the catalytic dwell, which results from ADP dissociation, while ATP-binding occurs at the catalytic dwell (Iida et al., 2019). It is noteworthy that ADP-dependent rotational backsteps are observed during *E. hirae* V_1_ power strokes, which are much larger than the small oscillations of the V_1_ΔHC power strokes observed here. Recent structures of the *T. thermophilus* A/V_1_, which also have the subunit D electrostatic interaction at the catalytic site that releases ADP indicate that ADP and Pi dissociate during the 120° power stroke, although at positions that are undefined to date (Figure 9B).

The V_1_ΔHC results presented here are consistent with MgATP binding to the empty catalytic site at 112°. The duration and occurrence of the 112° dwell increased when MgATP became rate-limiting, and large decreases in angular velocity were observed during the final 60° of rotation. Supporting further this conclusion, the duration of the 45° dwells did not increase with decreasing MgATP, even though the occurrence of these dwells increased to some extent. *E. hirae* V_1_ single-molecule studies suggested that ATP binding occurs during catalytic dwells (Iida et al., 2019). It is noteworthy that the S. *cerevisiae* V_1_ΔHC catalytic dwell duration did not change significantly when the major decrease in ATP hydrolysis occurred (from 490 μM to 5.7 μM MgATP). However, dwell duration did increase by 1.86-fold relative to 990 μM MgATP (Table 1) suggesting that some V_1_ΔHC molecules may bind MgATP during the catalytic dwell 8° later when MgATP is saturating.

The 112° dwell is unique in several ways. Its frequency of occurrence is MgATP dependent. The dwell occurrence is significantly more frequent when MgATP concentration is limiting at 5.7 μM (23.6% occurrence) than saturating at 990 μM (8.5% occurrence) (Table 2). In addition, the dwell is distinctly preceded by a major angular velocity reduction (Figure 8) that is remarkably steep at limiting MgATP. A nucleotide binding dwell 112° after the catalytic dwell has not been observed in other rotary ATPases to our knowledge. The eukaryotic V-ATPase is uniquely regulated by reversible disassembly of V_1_ and V_O_, which is an important regulatory mechanism that requires V_1_V_O_ ATP hydrolysis (Parra and Kane, 1998) that traps the dissociated V_1_ complex in a specific rotational state (Oot et al., 2016; Vasanthakumar et al., 2022). More work is required to determine whether this distinct 112° dwell is a functional adaptation of the eukaryotic rotary V-ATPases, and/or has been observed here as the result of increased resolution of our single-molecule assay.

## Scope statement

V-ATPases (V_1_V_O_-ATPases) are conserved rotary molecular motors that regulate cellular pH and play crucial roles in a large repertoire of physiological processes and human illnesses. Developing therapies that target V_1_V_O_-ATPase with precision requires understanding dynamics of V-ATPase rotation at high resolution at a molecular level. Here we report single-molecule rotation studies of the yeast V_1_-ATPase complex with high resolution of time and rotational position. Single molecules of V_1_-dependent rotation occurred in 120° power strokes similar to those of other rotary ATPases. However, these 120° rotational steps were interrupted by dwells at 45° and 112° when the product (ADP) was released and a new substrate (ATP) bound, respectively. This nucleotide binding sub-step at 112°, which may be unique to eukaryotic V_1_-ATPases, was distinctly preceded by a major reduction in angular velocity. This is important because current V-ATPase inhibitors immobilize the V_1_V_O_ assembled state, although V-ATPases are regulated by reversibly disassembling V_1_ and V_O_ *in vivo*. These results will help to design drugs that target a specific rotational sub-step to prevent reassembly and trap a disassembled and naturally inhibited state, leading the way in the development of a new generation of treatments that reversibly control V-ATPase function.

## Data Availability

The raw data supporting the conclusion of this article will be made available by the authors, without undue reservation.

## References

[B1] Alper S. L. (2010). Familial renal tubular acidosis. J. Nephrol. 23 (16), S57–S76. 21170890

[B2] Arai S. Saijo S. Suzuki K. Mizutani K. Kakinuma Y. Ishizuka-Katsura Y. (2013). Rotation mechanism of Enterococcus hirae V1-ATPase based on asymmetric crystal structures. Nature 493 (7434), 703–707. 10.1038/nature11778 23334411

[B3] Arsenieva D. Symersky J. Wang Y. Pagadala V. Mueller D. M. (2010). Crystal structures of mutant forms of the yeast F1 ATPase reveal two modes of uncoupling. J. Biol. Chem. 285 (47), 36561–36569. 10.1074/jbc.M110.174383 20843806 PMC2978584

[B4] Balakrishna A. M. Basak S. Manimekalai M. S. Gruber G. (2015). Crystal structure of subunits D and F in complex gives insight into energy transmission of the eukaryotic V-ATPase from *Saccharomyces cerevisiae* . J. Biol. Chem. 290 (6), 3183–3196. 10.1074/jbc.M114.622688 25505269 PMC4318993

[B5] Benlekbir S. Bueler S. A. Rubinstein J. L. (2012). Structure of the vacuolar-type ATPase from *Saccharomyces cerevisiae* at 11-A resolution. Nat. Struct. Mol. Biol. 19 (12), 1356–1362. 10.1038/nsmb.2422 23142977

[B6] Bilyard T. Nakanishi-Matsui M. Steel B. C. Pilizota T. Nord A. L. Hosokawa H. (2013). High-resolution single-molecule characterization of the enzymatic states in *Escherichia coli* F1-ATPase. Philos. Trans. R. Soc. Lond B Biol. Sci. 368 (1611), 20120023. 10.1098/rstb.2012.0023 23267177 PMC3538426

[B7] Boltz K. W. Frasch W. D. (2006). Hydrogen bonds between the alpha and beta subunits of the F1-ATPase allow communication between the catalytic site and the interface of the beta catch loop and the gamma subunit. Biochemistry 45 (37), 11190–11199. 10.1021/bi052592+ 16964980

[B8] Boyer P. D. (2002). Catalytic site occupancy during ATP synthase catalysis. FEBS Lett. 512 (1-3), 29–32. 10.1016/s0014-5793(02)02293-7 11852046

[B9] Breton S. Brown D. (2013). Regulation of luminal acidification by the V-ATPase. Physiol. (Bethesda) 28 (5), 318–329. 10.1152/physiol.00007.2013 PMC3768094 23997191

[B10] Collins M. P. Forgac M. (2020). Regulation and function of V-ATPases in physiology and disease. Biochim. Biophys. Acta Biomembr. 1862 (12), 183341. 10.1016/j.bbamem.2020.183341 32422136 PMC7508768

[B11] Cotter K. Liberman R. Sun-Wada G. Wada Y. Sgroi D. Naber S. (2016). The a3 isoform of subunit a of the vacuolar ATPase localizes to the plasma membrane of invasive breast tumor cells and is overexpressed in human breast cancer. Oncotarget 7 (29), 46142–46157. 10.18632/oncotarget.10063 27323815 PMC5216787

[B12] Cotter K. Stransky L. McGuire C. Forgac M. (2015). Recent insights into the structure, regulation, and function of the V-ATPases. Trends Biochem. Sci. 40 (10), 611–622. 10.1016/j.tibs.2015.08.005 26410601 PMC4589219

[B13] Diepholz M. Venzke D. Prinz S. Batisse C. Florchinger B. Rossle M. (2008). A different conformation for EGC stator subcomplex in solution and in the assembled yeast V-ATPase: possible implications for regulatory disassembly. Structure 16 (12), 1789–1798. 10.1016/j.str.2008.09.010 19081055

[B14] Drory O. Frolow F. Nelson N. (2004). Crystal structure of yeast V-ATPase subunit C reveals its stator function. EMBO Rep. 5 (12), 1148–1152. 10.1038/sj.embor.7400294 15540116 PMC1299189

[B15] Forgac M. (2007). Vacuolar ATPases: rotary proton pumps in physiology and pathophysiology. Nat. Rev. Mol. Cell Biol. 8 (11), 917–929. 10.1038/nrm2272 17912264

[B16] Frasch W. D. Bukhari Z. A. Yanagisawa S. (2022). F(1)F(O) ATP synthase molecular motor mechanisms. Front. Microbiol. 13, 965620. 10.3389/fmicb.2022.965620 36081786 PMC9447477

[B17] Furuike S. Nakano M. Adachi K. Noji H. Kinosita K. Jr. Yokoyama K. (2011). Resolving stepping rotation in Thermus thermophilus H(+)-ATPase/synthase with an essentially drag-free probe. Nat. Commun. 2, 233. 10.1038/ncomms1215 21407199 PMC3072102

[B18] Greene M. D. Frasch W. D. (2003). Interactions among gamma R268, gamma Q269, and the beta subunit catch loop of *Escherichia coli* F1-ATPase are important for catalytic activity. J. Biol. Chem. 278 (51), 51594–51598. 10.1074/jbc.M309948200 14532272

[B19] Hayek S. R. Lee S. A. Parra K. J. (2014). Advances in targeting the vacuolar proton-translocating ATPase (V-ATPase) for anti-fungal therapy. Front. Pharmacol. 5, 4. 10.3389/fphar.2014.00004 24478704 PMC3902353

[B20] Hayek S. R. Rane H. S. Parra K. J. (2019). Reciprocal regulation of V-ATPase and glycolytic pathway elements in Health and disease. Front. Physiol. 10, 127. 10.3389/fphys.2019.00127 30828305 PMC6384264

[B21] Hinton A. Bond S. Forgac M. (2009). V-ATPase functions in normal and disease processes. Pflugers Arch. 457 (3), 589–598. 10.1007/s00424-007-0382-4 18026982

[B22] Hirata T. Iwamoto-Kihara A. Sun-Wada G. H. Okajima T. Wada Y. Futai M. (2003). Subunit rotation of vacuolar-type proton pumping ATPase: relative rotation of the G and C subunits. J. Biol. Chem. 278 (26), 23714–23719. 10.1074/jbc.M302756200 12670943

[B23] Hirata T. Nakamura N. Omote H. Wada Y. Futai M. (2000). Regulation and reversibility of vacuolar H(+)-ATPase. J. Biol. Chem. 275 (1), 386–389. 10.1074/jbc.275.1.386 10617629

[B24] Iida T. Minagawa Y. Ueno H. Kawai F. Murata T. Iino R. (2019). Single-molecule analysis reveals rotational substeps and chemo-mechanical coupling scheme of Enterococcus hirae V1-ATPase. J. Biol. Chem. 294 (45), 17017–17030. 10.1074/jbc.RA119.008947 31519751 PMC6851342

[B25] Imamura H. Nakano M. Noji H. Muneyuki E. Ohkuma S. Yoshida M. (2003). Evidence for rotation of V1-ATPase. Proc. Natl. Acad. Sci. U. S. A. 100 (5), 2312–2315. 10.1073/pnas.0436796100 12598655 PMC151337

[B26] Ishmukhametov R. Hornung T. Spetzler D. Frasch W. D. (2010). Direct observation of stepped proteolipid ring rotation in *E. coli* F₀F₁-ATP synthase. EMBO J. 29 (23), 3911–3923. 10.1038/emboj.2010.259 21037553 PMC3020647

[B27] Jaskolka M. C. Tarsio M. Smardon A. M. Khan M. M. Kane P. M. (2021). Defining steps in RAVE-catalyzed V-ATPase assembly using purified RAVE and V-ATPase subcomplexes. J. Biol. Chem. 296, 100703. 10.1016/j.jbc.2021.100703 33895134 PMC8138766

[B28] Kane P. M. (1995). Disassembly and reassembly of the yeast vacuolar H(+)-ATPase *in vivo* . J. Biol. Chem. 270 (28), 17025–17032. 10.1016/s0021-9258(17)46944-4 7622524

[B29] Kane P. M. (2006). The where, when, and how of organelle acidification by the yeast vacuolar H+-ATPase. Microbiol. Mol. Biol. Rev. 70 (1), 177–191. 10.1128/MMBR.70.1.177-191.2006 16524922 PMC1393255

[B30] Kartner N. Manolson M. F. (2014). Novel techniques in the development of osteoporosis drug therapy: the osteoclast ruffled-border vacuolar H(+)-ATPase as an emerging target. Expert Opin. Drug Discov. 9 (5), 505–522. 10.1517/17460441.2014.902155 24749538

[B31] Kawasaki-Nishi S. Nishi T. Forgac M. (2001). Arg-735 of the 100-kDa subunit a of the yeast V-ATPase is essential for proton translocation. Proc. Natl. Acad. Sci. U. S. A. 98 (22), 12397–12402. 10.1073/pnas.221291798 11592980 PMC60065

[B32] Kayalar C. Rosing J. Boyer P. D. (1977). An alternating site sequence for oxidative phosphorylation suggested by measurement of substrate binding patterns and exchange reaction inhibitions. J. Biol. Chem. 252 (8), 2486–2491. 10.1016/s0021-9258(17)40484-4 856791

[B33] Khan M. M. Lee S. Couoh-Cardel S. Oot R. A. Kim H. Wilkens S. (2022). Oxidative stress protein Oxr1 promotes V-ATPase holoenzyme disassembly in catalytic activity-independent manner. EMBO J. 41 (3), e109360. 10.15252/embj.2021109360 34918374 PMC8804929

[B34] Kobayashi R. Ueno H. Li C. B. Noji H. (2020). Rotary catalysis of bovine mitochondrial F(1)-ATPase studied by single-molecule experiments. Proc. Natl. Acad. Sci. U. S. A. 117 (3), 1447–1456. 10.1073/pnas.1909407117 31896579 PMC6983367

[B35] Licon-Munoz Y. Fordyce C. A. Hayek S. R. Parra K. J. (2018). V-ATPase-dependent repression of androgen receptor in prostate cancer cells. Oncotarget 9 (48), 28921–28934. 10.18632/oncotarget.25641 29988966 PMC6034745

[B36] Lotscher H. R. deJong C. Capaldi R. A. (1984). Interconversion of high and low adenosinetriphosphatase activity forms of *Escherichia coli* F1 by the detergent lauryldimethylamine oxide. Biochemistry 23 (18), 4140–4143. 10.1021/bi00313a020 6237684

[B37] Martin J. L. Ishmukhametov R. Hornung T. Ahmad Z. Frasch W. D. (2014). Anatomy of F1-ATPase powered rotation. Proc. Natl. Acad. Sci. U. S. A. 111 (10), 3715–3720. 10.1073/pnas.1317784111 24567403 PMC3956197

[B38] Martin J. L. Ishmukhametov R. Spetzler D. Hornung T. Frasch W. D. (2018). Elastic coupling power stroke mechanism of the F1-ATPase molecular motor. Proc. Natl. Acad. Sci. U. S. A. 115 (22), 5750–5755. 10.1073/pnas.1803147115 29760063 PMC5984535

[B39] Mazhab-Jafari M. T. Rohou A. Schmidt C. Bueler S. A. Benlekbir S. Robinson C. V. (2016). Atomic model for the membrane-embedded V(O) motor of a eukaryotic V-ATPase. Nature 539 (7627), 118–122. 10.1038/nature19828 27776355 PMC7332345

[B40] Minagawa Y. Ueno H. Hara M. Ishizuka-Katsura Y. Ohsawa N. Terada T. (2013). Basic properties of rotary dynamics of the molecular motor Enterococcus hirae V1-ATPase. J. Biol. Chem. 288 (45), 32700–32707. 10.1074/jbc.M113.506329 24089518 PMC3820904

[B41] Muench S. P. Trinick J. Harrison M. A. (2011). Structural divergence of the rotary ATPases. Q. Rev. Biophys. 44 (3), 311–356. 10.1017/S0033583510000338 21426606

[B42] Oot R. A. Couoh-Cardel S. Sharma S. Stam N. J. Wilkens S. (2017). Breaking up and making up: the secret life of the vacuolar H(+) -ATPase. Protein Sci. 26 (5), 896–909. 10.1002/pro.3147 28247968 PMC5405435

[B43] Oot R. A. Huang L. S. Berry E. A. Wilkens S. (2012). Crystal structure of the yeast vacuolar ATPase heterotrimeric EGC(head) peripheral stalk complex. Structure 20 (11), 1881–1892. 10.1016/j.str.2012.08.020 23000382 PMC3496068

[B44] Oot R. A. Kane P. M. Berry E. A. Wilkens S. (2016). Crystal structure of yeast V1-ATPase in the autoinhibited state. EMBO J. 35 (15), 1694–1706. 10.15252/embj.201593447 27295975 PMC4969575

[B45] Parra K. J. Chan C. Y. Chen J. (2014). *Saccharomyces cerevisiae* vacuolar H+-ATPase regulation by disassembly and reassembly: one structure and multiple signals. Eukaryot. Cell 13 (6), 706–714. 10.1128/EC.00050-14 24706019 PMC4054264

[B46] Parra K. J. Kane P. M. (1998). Reversible association between the V1 and V0 domains of yeast vacuolar H+-ATPase is an unconventional glucose-induced effect. Mol. Cell Biol. 18 (12), 7064–7074. 10.1128/MCB.18.12.7064 9819393 PMC109288

[B47] Parra K. J. Keenan K. L. Kane P. M. (2000). The H subunit (Vma13p) of the yeast V-ATPase inhibits the ATPase activity of cytosolic V1 complexes. J. Biol. Chem. 275 (28), 21761–21767. 10.1074/jbc.M002305200 10781598

[B48] Ragunathan P. Sielaff H. Sundararaman L. Biukovic G. Subramanian Manimekalai M. S. Singh D. (2017). The uniqueness of subunit α of mycobacterial F-ATP synthases: an evolutionary variant for niche adaptation. J. Biol. Chem. 292 (27), 11262–11279. 10.1074/jbc.M117.784959 28495884 PMC5500794

[B49] Roh S. H. Stam N. J. Hryc C. F. Couoh-Cardel S. Pintilie G. Chiu W. (2018). The 3.5-A CryoEM structure of nanodisc-reconstituted yeast vacuolar ATPase V(o) proton channel. Mol. Cell 69 (6), 993–1004. 10.1016/j.molcel.2018.02.006 29526695 PMC5893162

[B50] Sagermann M. Stevens T. H. Matthews B. W. (2001). Crystal structure of the regulatory subunit H of the V-type ATPase of *Saccharomyces cerevisiae* . Proc. Natl. Acad. Sci. U. S. A. 98 (13), 7134–7139. 10.1073/pnas.131192798 11416198 PMC34635

[B51] Schep D. G. Zhao J. Rubinstein J. L. (2016). Models for the a subunits of the Thermus thermophilus V/A-ATPase and *Saccharomyces cerevisiae* V-ATPase enzymes by cryo-EM and evolutionary covariance. Proc. Natl. Acad. Sci. U. S. A. 113 (12), 3245–3250. 10.1073/pnas.1521990113 26951669 PMC4812769

[B52] Sielaff H. Martin J. Singh D. Biukovic G. Gruber G. Frasch W. D. (2016). Power stroke angular velocity profiles of archaeal A-ATP synthase versus thermophilic and mesophilic F-ATP synthase molecular motors. J. Biol. Chem. 291 (49), 25351–25363. 10.1074/jbc.M116.745240 27729450 PMC5207238

[B53] Sobti M. Ueno H. Noji H. Stewart A. G. (2021). The six steps of the complete F(1)-ATPase rotary catalytic cycle. Nat. Commun. 12 (1), 4690. 10.1038/s41467-021-25029-0 34344897 PMC8333055

[B54] Sobti M. Walshe J. L. Wu D. Ishmukhametov R. Zeng Y. C. Robinson C. V. (2020). Cryo-EM structures provide insight into how *E. coli* F(1)F(o) ATP synthase accommodates symmetry mismatch. Nat. Commun. 11 (1), 2615. 10.1038/s41467-020-16387-2 32457314 PMC7251095

[B55] Spetzler D. Ishmukhametov R. Hornung T. Day L. J. Martin J. Frasch W. D. (2009). Single molecule measurements of F1-ATPase reveal an interdependence between the power stroke and the dwell duration. Biochemistry 48 (33), 7979–7985. 10.1021/bi9008215 19610671 PMC2737049

[B56] Spetzler D. York J. Daniel D. Fromme R. Lowry D. Frasch W. (2006). Microsecond time scale rotation measurements of single F1-ATPase molecules. Biochemistry 45 (10), 3117–3124. 10.1021/bi052363n 16519506 PMC4494661

[B57] Sumner J. P. Dow J. A. Earley F. G. Klein U. Jager D. Wieczorek H. (1995). Regulation of plasma membrane V-ATPase activity by dissociation of peripheral subunits. J. Biol. Chem. 270 (10), 5649–5653. 10.1074/jbc.270.10.5649 7890686

[B58] Suzuki K. Mizutani K. Maruyama S. Shimono K. Imai F. L. Muneyuki E. (2016). Crystal structures of the ATP-binding and ADP-release dwells of the V(1) rotary motor. Nat. Commun. 7, 13235. 10.1038/ncomms13235 27807367 PMC5095293

[B59] Suzuki T. Tanaka K. Wakabayashi C. Saita E. Yoshida M. (2014). Chemomechanical coupling of human mitochondrial F1-ATPase motor. Nat. Chem. Biol. 10 (11), 930–936. 10.1038/nchembio.1635 25242551

[B60] Vasanthakumar T. Bueler S. A. Wu D. Beilsten-Edmands V. Robinson C. V. Rubinstein J. L. (2019). Structural comparison of the vacuolar and Golgi V-ATPases from *Saccharomyces cerevisiae* . Proc. Natl. Acad. Sci. U. S. A. 116 (15), 7272–7277. 10.1073/pnas.1814818116 30910982 PMC6462096

[B61] Vasanthakumar T. Keon K. A. Bueler S. A. Jaskolka M. C. Rubinstein J. L. (2022). Coordinated conformational changes in the V(1) complex during V-ATPase reversible dissociation. Nat. Struct. Mol. Biol. 29 (5), 430–439. 10.1038/s41594-022-00757-z 35469063

[B62] Wang R. Long T. Hassan A. Wang J. Sun Y. Xie X. S. (2020). Cryo-EM structures of intact V-ATPase from bovine brain. Nat. Commun. 11 (1), 3921. 10.1038/s41467-020-17762-9 32764564 PMC7414150

[B63] Yasuda R. Noji H. Yoshida M. Kinosita K. Jr. Itoh H. (2001). Resolution of distinct rotational substeps by submillisecond kinetic analysis of F1-ATPase. Nature 410 (6831), 898–904. 10.1038/35073513 11309608

[B64] Yokoyama K. Nakano M. Imamura H. Yoshida M. Tamakoshi M. (2003). Rotation of the proteolipid ring in the V-ATPase. J. Biol. Chem. 278 (27), 24255–24258. 10.1074/jbc.M303104200 12707282

[B65] Zarco-Zavala M. Watanabe R. McMillan D. G. G. Suzuki T. Ueno H. Mendoza-Hoffmann F. (2020). The 3 × 120° rotary mechanism of Paracoccus denitrificans F1-ATPase is different from that of the bacterial and mitochondrial F1-ATPases. Proc. Natl. Acad. Sci. U. S. A. 117 (47), 29647–29657. 10.1073/pnas.2003163117 33168750 PMC7703542

[B66] Zhang Z. Charsky C. Kane P. M. Wilkens S. (2003). Yeast V1-ATPase: affinity purification and structural features by electron microscopy. J. Biol. Chem. 278 (47), 47299–47306. 10.1074/jbc.M309445200 12960158

[B67] Zhang Z. Zheng Y. Mazon H. Milgrom E. Kitagawa N. Kish-Trier E. (2008). Structure of the yeast vacuolar ATPase. J. Biol. Chem. 283 (51), 35983–35995. 10.1074/jbc.M805345200 18955482 PMC2602884

[B68] Zhao J. Benlekbir S. Rubinstein J. L. (2015). Electron cryomicroscopy observation of rotational states in a eukaryotic V-ATPase. Nature 521 (7551), 241–245. 10.1038/nature14365 25971514

